# Elevated ApoB/ApoA-I ratio is associated with acute anti-N-Methyl-D-aspartate receptor encephalitis, but not disease outcomes

**DOI:** 10.3389/fneur.2022.896656

**Published:** 2022-09-01

**Authors:** Yingying Liu, Xiaomeng Ma, Lili Ma, Zhumin Su, Donghong Li, Xiaohong Chen

**Affiliations:** Department of Neurology, Third Affiliated Hospital, Sun Yat-sen University, Guangzhou, China

**Keywords:** ApoB/ApoA-I ratio, anti-N-Methyl-D-aspartate receptor encephalitis, immune-inflammatory, disease outcomes, peripheral blood

## Abstract

**Objective:**

The purpose of the present study is to clarify the relationship between the apolipoprotein B100/apolipoprotein A-I (ApoB/ApoA-I) ratio and anti-N-methyl-D-aspartate receptor (anti-NMDAR) encephalitis.

**Methods:**

A total of 71 patients with anti-NMDAR encephalitis were included in this study, and their ApoB/ApoA-I ratios in baseline and follow-up were retrospectively analyzed.

**Results:**

The ApoB/ApoA-I ratio was closely correlated with the baseline-modified Rankin scale (mRS) score of >3 in patients with anti-NMDAR encephalitis. A subgroup analysis showed obvious differences between the high and low ApoB/ApoA-I ratio groups. The ApoB/ApoA-I ratio was positively correlated with intensive care unit (ICU) treatment, length of hospital stay, baseline mRS score, C-reactive protein (CRP), and erythrocyte sedimentation rate (ESR). The ratios of the high and low ApoB/ApoA-I groups both improved in the follow-up.

**Conclusion:**

The increased ApoB/ApoA-I ratio is associated with acute anti-NMDAR encephalitis, but not disease outcomes. Serum ApoB/ApoA-I ratio was related to inflammation and immunity in peripheral blood. The findings might provide a new idea for further exploration of the pathogenesis and treatment of anti-NMDAR encephalitis.

## Introduction

Anti-N-methyl-D-aspartate receptor (NMDAR) encephalitis is identified as a neurological autoimmune disorder associated with intrathecally synthesized antibodies against NR1–NR2 heteromers of the NMDA receptor. Patients with anti-NMDAR frequently present with a characteristic neuropsychiatric syndrome, such as psychosis problems, dyskinesia, and seizures (1, 2). They often eventually improve after early and positive immunotherapy, intensive care support, and tumor removal. However, its mortality rate has been reported to be 8–10% (1, 3). Previous studies have suggested that the long-term outcome of patients with severe anti-NMDAR encephalitis is difficult to predict (4, 5). Individuals with severe anti-NMDAR encephalitis generally need intensive care support, longer hospitalization periods, and more complex treatments (6). Furthermore, the population with anti-NMDAR encephalitis demonstrated poor long-term psychosocial function, high care transitions and caregiver burden, and heavy financial burden (7–9). The pathogenesis of this disease is awaited to clear, and potential biomarkers to indicate the disease development are needed to be explored.

Apolipoprotein B100 (ApoB) and Apolipoprotein A-I (ApoA-I) are two major blood lipoproteins. ApoB is a structural protein for low-density lipoprotein cholesterol (LDL-C), very low-density lipoprotein (VLDL), and intermediate density lipoprotein (IDL) (10). ApoA-I is the main apolipoprotein mostly existing in high-density lipoprotein cholesterol (HDL-C). ApoB transports lipids from the liver and intestine to the location of utilization, whereas ApoA-I mediates the reverse transport of cholesterol from peripheral tissues to the liver (10). It has been widely believed that ApoB particles are atherogenic, and ApoA-I exerts anti-atherogenic, antioxidant, anti-inflammatory, and anti-apoptotic effects. Therefore, the ApoB/ApoA-I ratio represents the balance between atherogenic and anti-atherosclerotic particles (11). Many clinical studies have demonstrated that ApoB/ApoA-I is a quite good predictive indicator for disease severity and prognosis. It is independently associated with acute coronary syndrome, diabetes, aortic stenosis, cancer, insulin resistance, and carotid intima media thickness (12–17). Similarly, ApoB, ApoA-I, and ApoB/ApoA-I ratios are also associated with many neurological diseases and immune-inflammatory diseases. ApoB is associated with the future risk of Parkinson's disease (PD) (18). Increased ApoA-I is related to a lower rate of cortical and gray matter loss in multiple sclerosis (MS) (19). The ApoB/ApoA-I ratio significantly increases in behavioral variant frontotemporal dementia (bvFTD) compared to controls (20). An increase in ApoB/ApoA-I ratio was associated with an increase in disease severity of psoriatic arthritis (PSA) and acute pancreatitis (AP) (21, 22). Therefore, we proposed a hypothesis that ApoB/ApoA-I ratio was associated with anti-NMDAR encephalitis, which was a neurological autoimmune disorder. We explored the association between ApoB/ApoA-I ratio and anti-NMDAR encephalitis in the present study.

We retrospectively analyzed 71 patients with anti-NMDAR encephalitis hospitalized in the Third Affiliated Hospital of Sun Yat-sen University from January 2015 to May 2019. The ApoB/ApoA-I ratio in baseline and follow-up of patients with anti-NMDAR encephalitis were analyzed and compared with those in the healthy controls. We searched the risk factors of patients with serious conditions and explored the correlation between them and the ApoB/ApoA-I ratio. The differences between the high and low ApoB/ApoA-I ratio groups were also compared. We explored the relationships between the ApoB/ApoA-I ratio and anti-NMDAR encephalitis, which may contribute to further studies in the pathogenesis and therapies of the disease.

## Materials and methods

### Study design and participants

We retrospectively analyzed patients with anti-NMDAR encephalitis hospitalized in the Third Affiliated Hospital of Sun Yat-sen University. The diagnosis of anti-NMDAR encephalitis met the criteria established in 2016 (23). The patients presented neurological symptoms, such as psychiatric symptoms, cognitive impairment, memory deficits, seizures, movement disorder or dyskinesia, speech dysfunction, disturbance of consciousness, central hypopnea, autonomic dysfunction, and so on. At the same time, the anti-NMDAR antibody was positive in cerebrospinal fluid (CSF). The inclusion criteria were as follows: (1) patients who had newly diagnosed anti-NMDAR encephalitis and (2) aged between 18 and 65 years; The exclusion criteria were: (1) patients who had a history or suffered from cardiovascular and cerebrovascular diseases, diabetes, neuromyelitis optic spectrum disease, MS, and blood diseases; (2) they showed lung or urinary tract infection or severe impairment of liver or kidney function; (3) they received hypolipidemic drugs or had a history of familial dyslipidemia; and (4) they had other autoimmune encephalitis antibody positive in serum or CSF.

Based on the inclusion and exclusion criteria, two trained researchers, respectively, searched and screened the study subjects in the electronic medical record database of the Third Affiliated Hospital of Sun Yat-sen University. The consistent subjects were enrolled in this study. We involved a total of 71 patients with anti-NMDAR encephalitis in the study. Their baseline and follow-up data were retrospectively analyzed. In the baseline period, demographic data, clinical manifestation, treatment, blood, and CSF testing data were collected. After the first discharge, patients were followed up for 3 months, and relevant serum tests were performed. In addition, the blood testing data of 71 healthy controls (CTLs) age-and sex-matched with the patients with anti-NMDAR encephalitis were collected from the physical examination center of our hospital.

### Biochemical assays

Patients underwent a modified Rankin scale (mRS) score evaluation, blood tests, CSF test, a brain magnetic resonance imaging (MRI) study, and an abdominal computed tomography (CT) scan or ultrasound as soon as possible after admission at baseline. mRS was used to evaluate the neurological status of patients with anti-NMDAR encephalitis (24). The mRS score ranges from 0 to 5. A higher score is associated with worse neurological functions, and the mRS score of >3 (4–5 scores) was defined as severe disability. Serum levels of lipid and lipoprotein such as total cholesterol (CHOL), triglyceride (TG), LDL-C, HDL-C, ApoA-I, ApoB, immunity factors such as C-reactive protein (CRP), immunoglobulin G (IgG), immunoglobulin A (IgA), immunoglobulin M (IgM), complement 3 (C3), complement 4 (C4), serum total complement (CH50), and CSF protein, glucose, and chloride were quantified by a direct enzymatic method on a Clinical Analyzer 7180-ISE (Hitachi High-Technologies, Japan). The level of non-HDL-C was CHOL minus HDL-C. CSF samples of all patients were analyzed by tissue-based assay (TBA) and cell-based assay (CBA). Anti-NMDAR IgG antibody was tested by indirect immunostaining using a detection kit (EUROIMMUN Medizinische Labordiagnostika, Lübeck, Germany). Abdominal CT scan or ultrasound was performed to detect teratoma.

### Treatment

Treatment included immunotherapies, tumor removal, and symptomatic and supportive therapy. All patients with anti-NMDAR encephalitis received the standard treatment (25, 26). Once the diagnosis was established during hospitalization, all patients started immediately first-line immunotherapies such as high-dose methylprednisolone (1.0 g/day for 3–7 days, intravenous injection) or/and intravenous immunoglobulin (0.4 g/kg/day for 3–5 days, intravenous injection). If there was no response to first-line therapy after 2–4 weeks, the second-line agent was added. There were 12 patients who received second-line immunotherapies, 7 for rituximab (375 mg/m^2^ body surface area, once a week for 3–4 weeks, intravenous injection), and 5 for cyclophosphamide (750 mg/m^2^ body surface area, once every 4 weeks, intravenous injection). All patients received low-dose oral prednisone (1 mg/kg/day, then at less dosage after stabilization of condition) since they were discharged from the hospital. Long-term immunosuppression was considered in patients with severe initial presentation or high recurrence rate (e.g., a tumor was not found). Long-term immunosuppression was added to 13 patients, 2 for mycophenolate mofetil (1,000–2,000 mg/day, oral administration), 8 for azathioprine (100 mg/day, oral administration), and 3 for cyclosporine (3–5 mg/kg/day, oral administration). Ovarian teratoma resection was started when a paraneoplastic etiology was confirmed.

### Ethics approval statement

The present study involving human participants was reviewed and approved by the Ethics Committee of the Third Affiliated Hospital of Sun Yat-sen University. Because the subjects could not be contacted, this study was exempted from informed consent and approved by the ethics committee of the Third Affiliated Hospital of Sun Yat-sen University.

### Statistical analysis

Statistical Package for the Social Sciences (SPSS) 25.0 software (IBM, Chicago, IL, USA) was used for statistical analyses. Continuous variables were described as mean ± standard deviation (SD) or median, whereas categorical variables were described as percentages. The independent *t*-test and Mann–Whitney *U*-test were used for comparison of continuous variables and Pearson's chi-squared for categorical variables between the two groups. A one-way analysis of variance (ANOVA) was used for multiple comparisons, followed by the least significant difference (LSD) and Tamhane T2(M) were performed for *post-hoc* test. Logistic regression tests were conducted to calculate odds ratios (ORs) and 95% confidence intervals (CIs) for mRS score of >3 in patients with anti-NMDAR encephalitis. Correlation analysis was carried out with Pearson's and Spearman's correlation. A two-sided *p*-value of <0.05 was defined as statistically significant.

## Results

### Patients with anti-NMDAR encephalitis had significantly higher ApoB/ApoA-I ratio, platelet (PLT)/HDL-C, and neutrophil/HDL-C

A total of 71 patients with anti-NMDAR encephalitis and 71 CTLs were included in this study. The median age of the patients was 33 years, and 47.89% of patients were women, and there was no significant difference in age and sex between CTL and patient groups. The baseline clinical characteristics and laboratory parameters of individuals are shown in Table 1. Patients with anti-NMDAR encephalitis had significantly higher neutrophil (*p* < 0.001), ApoB/ApoA-I (*p* < 0.001), PLT/HDL-C (*p* = 0.039), and neutrophil/HDL-C (*p* < 0.001) compared to CTLs. The levels of TC (*p* = 0.042), HDL-C (*p* = 0.02), and ApoA-I (*p* < 0.001) were significantly lower in patients with anti-NMDAR encephalitis compared to CTLs. In addition, PLT, CHOL, LDL-C, ApoB, non-HDL-C, and non-HDL-C/HDL-C had no difference between the two groups. The changes in ApoA-I, ApoB, and ApoB/ApoA-I ratios were independent of gender (Table 1).

**Table 1:** Baseline characteristics of patients with anti-NMDAR encephalitis and healthy controls.

	**Patients with anti-NMDAR encephalitis (*n* = 71)**	**CTLs (*n* = 71)**	***p***
Age (years)	33	35	0.556
Female (%)	47.89	47.89	1.000
Psychiatric symptoms (%)	90.14	–	–
Seizures (%)	61.97	–	–
Disturbance of consciousness (%)	26.76	–	–
Central hypopnea (%)	14.08	–	–
Baseline mRS score of >3 (%)	38.03	–	–
Accompanied teratoma (%)	14.08	–	–
Abnormal brain MRI (%)	39.44	–	–
ICU treatment (%)	19.72	–	–
Length of hospital stay (days)	24	–	–
PLT (10^9^/L)	254.49 ± 83.74	263.42 ± 56.79	0.459
Neutrophil (10^9^/L)	7.18 ± 3.97	3.84 ± 1.57	<0.001^**^
CHOL (mmol/L)	4.41 ± 0.94	4.71 ± 0.81	0.042^*^
TG (mmol/L)	1.13 ± 0.51	1.05 ± 0.46	0.324
LDL-C (mmol/L)	2.84 ± 0.85	2.98 ± 0.74	0.278
HDL-C (mmol/L)	1.16 ± 0.43	1.31 ± 0.31	0.02^*^
**ApoA-I (g/L)**	1.12 ± 0.28	1.47 ± 0.22	<0.001^**^
Male	1.10 ± 0.30	1.45 ± 0.24	<0.001^**^
Female	1.14 ± 0.26	1.49 ± 0.20	<0.001^**^
**ApoB (g/L)**	0.87 ± 0.28	0.87 ± 0.20	0.960
Male	0.84 ± 0.29	0.90 ± 0.21	0.346
Female	0.90 ± 0.27	0.83 ± 0.20	0.262
Non-HDL-C	3.25 ± 0.86	3.40 ± 0.79	0.267
**ApoB/ApoA-I (ratio)**	0.83 ± 0.37	0.60 ± 0.17	<0.001^**^
Male	0.84 ± 0.41	0.64 ± 0.18	0.012^*^
Female	0.83 ± 0.32	0.57 ± 0.15	<0.001^**^
Non-HDL-C/HDL-C (ratio)	3.13 ± 1.41	2.75 ± 0.95	0.06
PLT/HDL-C (ratio)	241.96 ± 106.29	210.97 ± 65.32	0.039^*^
Neutrophil /HDL-C (ratio)	6.99 ± 4.66	3.11 ± 1.44	<0.001^**^
LDL-C/HDL-C (ratio)	2.69 ± 1.15	2.40 ± 0.84	0.092

Continuous variables with were described as mean ± standard deviation or median, whereas categorical variables as percentages. The independent *t*-test and the Mann–Whitney *U*-test were used for comparison of continuous variables and Pearson's chi-squared test for categorical variables between the two groups. A two-sided *p* < 0.05 was considered significant. anti-NMDAR, anti-N-methyl-D-aspartate receptor; CTLs, controls; mRS, modified Rankin scale; MRI, brain magnetic resonance imaging; ICU, intensive care unit; PLT, platelet; CHOL, total cholesterol; TG, triglyceride; LDL-C, low-density lipoprotein cholesterol; HDL-C, high-density lipoprotein cholesterol; ApoA-I, apolipoprotein A-I; ApoB, apolipoprotein B100.

* *p* < 0.05,

** *p* < 0.001.

### The ApoB/ApoA-I ratio was closely correlated with the baseline mRS score of >3 in patients with anti-NMDAR encephalitis

**Figure 1:**
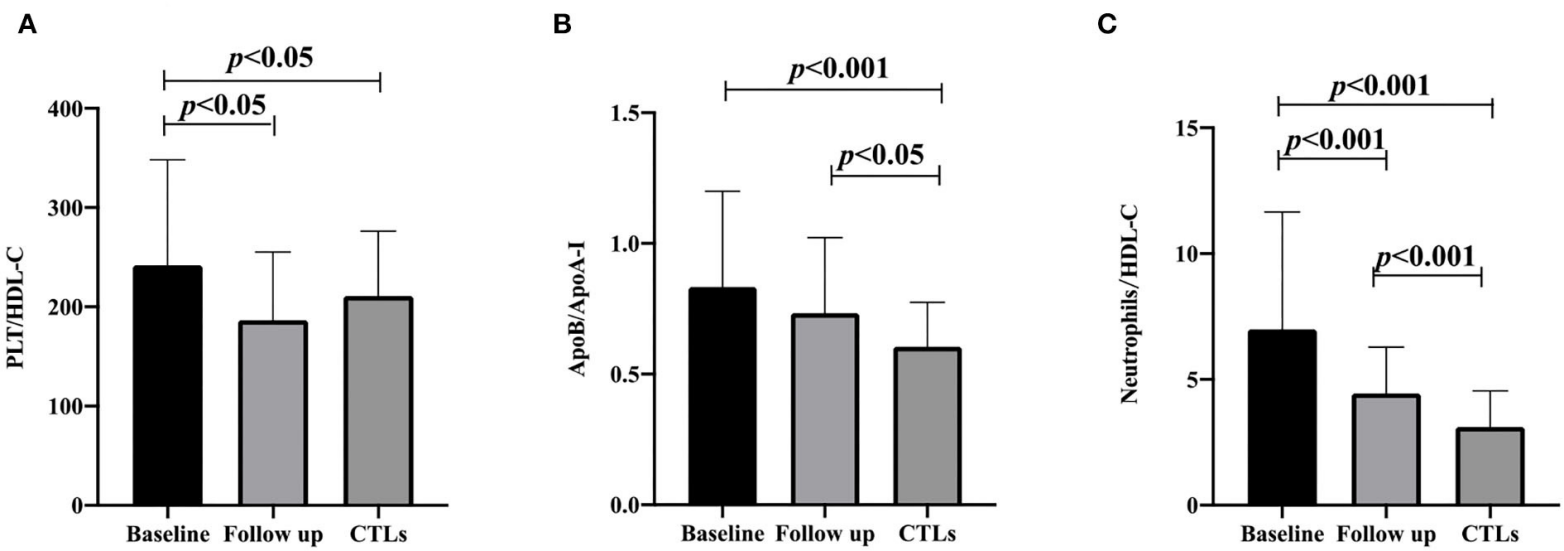
PLT/HDL-C ratio, ApoB/ApoA-I ratio, and neutrophils/HDL-C ratio are compared with CTLs at the baseline and follow-up of anti-NMDAR encephalitis. **(A)** PLT/HDL-C ratio; **(B)** ApoB/ApoA-I ratio; **(C)** neutrophils/HDL-C ratio. A one-way analysis of variance (ANOVA) was used for multiple comparisons, following by the least significant difference (LSD) and Tamhane T2(M) were performed for *post-hoc* test. A two-sided *p*-value of <0.05 was defined statistically significant. CTLs, controls; PLT, platelet; HDL-C, high-density lipoprotein cholesterol; ApoA-I, apolipoprotein A-I; ApoB, apolipoprotein B100.

After a 3-month follow-up, PLT/HDL-C and neutrophil/HDL-C were significantly decreased compared to baseline (186.65 ± 68.64 vs. 241.96 ± 106.29 *p* = 0.001, and 4.45 ± 1.83 vs. 6.99 ± 4.66 *p* < 0.001), and the ApoB/ApoA-I ratio did not change significantly from the baseline (0.73 ± 0.29 vs. 0.83 ± 0.37 *p* = 0.142). Compared with that in the CTL group, the ApoB/ApoA-I and neutrophil/HDL-C ratios increased after follow-up (0.60 ± 0.17 vs. 0.73 ± 0.29 *p* = 0.013, and 3.11 ± 1.44 vs. 4.45 ± 1.83 *p* < 0.001). PLT/HDL-C was comparable between the CTL group and the follow-up patient group (210.97 ± 65.32 vs. 186.65 ± 68.64 *p* = 0.061; Figure 1). Furthermore, multivariate logistic regression analysis found that the ApoB/ApoA-I ratio was determined to be closely correlated with a baseline mRS score of >3 in patients with anti-NMDAR encephalitis, but not PLT/HDL-C and neutrophil/HDL-C (Table 2).

**Table 2:** Logistic regression analysis of factors related to mRS score of >3 in patients with anti-NMDAR encephalitis patients.

**Variables**	**B**	**SE**	**Exp (B)**	**95% CI for Exp (B)**	***p***
Age	0.015	0.029	1.016	0.960–1.074	0.589
Female	1.195	0.626	3.303	0.969–11.257	0.056
PLT/HDL-C	0.006	0.004	1.006	0.999–1.013	0.107
ApoB/ApoA-I	1.959	0.942	7.093	1.119–44.968	0.038^*^
Neutrophil /HDL-C	0.001	0.075	1.001	0.863–1.160	0.994

A two-sided *p*-value of <0.05 was defined statistically significant. PLT, platelet; HDL-C, high-density lipoprotein cholesterol; ApoA-I, apolipoprotein A-I; ApoB, apolipoprotein B100.

* *p* < 0.05,

** *p* < 0.001.

### The ApoB/ApoA-I ratio was positively correlated with ICU treatment, length of hospital stay, baseline mRS score, CRP, and ESR

Patients with anti-NMDAR encephalitis were divided into two groups according to the ApoB/ApoA-I ratio. The ApoB/ApoA-I ratio cutoff value for high vs. low subgroup analysis was determined to be 0.6, based on a reference sample from the FINRISK 2007 study (27) and the average of CTLs in the present study. The high group was with ApoB/ApoA-I higher than 0.6 (1.00 ± 0.31, *n* = 45), whereas the low group was <0.6 (0.46 ± 0.11, *n* = 22), and four cases with obvious deviation were excluded. Subgroup analysis showed that there were many differences between the high and low ApoB/ApoA-I ratio groups. The high ApoB/ApoA-I group patients had significantly worse psychiatric symptoms (*p* = 0.034), longer length of ICU stay (*p* = 0.044), longer length of hospital stay (*p* = 0.017), and higher levels of CRP (*p* = 0.018), IgG (*p* = 0.013), C3 (*p* = 0.021), and ESR (*p* = 0.042) compared to the low ApoB/ApoA-I group. The age, gender, seizures, disturbance of consciousness, central hypopnea, accompanied teratoma, abnormal brain MR, ICU treatment, second-line immunotherapies, long-term immunosuppression, treatment delay of >4 weeks, and lack of clinical improvement within 4 weeks had no difference between the two groups. IgA, IgM, C4, CH50, CSF pressure, CSF red blood cells (RBCs), CSF white blood cells (WBCs), CSF protein, CSF glucose, and CSF chloride also showed no difference between the two groups (Table 3). Furthermore, correlation analysis showed that the ApoB/ApoA-I ratio (*r* = 0.303, *p* = 0.010) was positively correlated with ICU treatment, length of hospital stay, CRP, ESR, and baseline mRS score of >3 in patients with anti-NMDAR encephalitis (Table 4).

**Table 3:** Baseline characteristics of high and low ApoB/ApoA-I ratio in patients with anti-NMDAR encephalitis.

	**ApoB/ApoA-I (ratio)**		***p***
	**High (>0.60, *n* = 45)**	**Low ( ≤ 0.60, *n* = 22)**	
ApoB/ApoA-I (ratio)	1.00 ± 0.31	0.46 ± 0.11	<0.001^**^
Age (years)	31	24	0.075
Female (%)	51.11	45.45	0.083
Psychiatric symptoms (%)	95.56	77.27	0.034^*^
Seizures (%)	60.00	63.64	0.774
Disturbance of consciousness (%)	31.11	22.73	0.475
Central hypopnea (%)	17.78	9.09	0.567
Accompanied teratoma (%)	17.50	16.67	0.938
Abnormal brain MR (%)	47.50	42.11	0.698
ICU treatment (%)	24.44	9.09	0.245
Length of ICU stay (days)	16	4	0.044^*^
Length of hospital stay (days)	28.00	14.00	0.017^*^
Second-line immunotherapies (*n*)	7	5	0.472
Long-term immunosuppression (*n*)	9	3	0.765
Treatment delay >4 weeks (*n*)	24	7	0.097
Lack of clinical improvement within 4 weeks (*n*)	11	3	0.483
CRP (mg/L)	4.05	2.38	0.018^*^
IgG (g/L)	13.19	10.5	0.013^*^
IgA (g/L)	2.20	2.24	0.481
IgM (g/L)	1.04 ± 0.45	1.03 ± 0.65	0.955
C3 (g/L)	1.19 ± 0.21	1.04 ± 0.19	0.021^*^
C4 (g/L)	0.25	0.25	0.896
CH50 (U/ml)	50.70 ± 14.44	43.89 ± 14.67	0.253
ESR (mm/H)	16.50	7.50	0.042^*^
CSF pressure (mmH_2_O)	164.83 ± 56.39	156.88 ± 34.54	0.602
CSF RBC (10^6^/L)	2.00	2.00	0.972
CSF WBC (10^6^/L)	4.50	6.00	0.779
CSF protein (g/L)	0.27	0.23	0.599
CSF glucose (mmol/L)	3.57	3.57	0.640
CSF chloride (mmol/L)	123.10	123.4	0.441

Continuous variables with were described as mean ± standard deviation or median, whereas categorical variables as *n* or percentages. The independent *t*-test and Mann–Whitney *U*-test were used for comparison of continuous variables and Pearson's chi-squared test for categorical variables between the two groups. A two-sided *p* < 0.05 was considered significant. anti-NMDAR, anti-N-methyl-D-aspartate receptor; ApoA-I, apolipoprotein A-I; ApoB, apolipoprotein B100; MRI, brain magnetic resonance imaging; ICU, intensive care unit; CRP, C-reactive protein; IgG, immunoglobulin G; IgA, immunoglobulin A; IgM, immunoglobulin M; C3, complement 3; C4, complement 4; CH50, serum total complement; ESR, erythrocyte sedimentation rate; CSF, cerebrospinal fluid; RBC, red blood cells; WBC, white blood cells.

* *p* < 0.05,

** *p* < 0.001.

**Table 4:** Correlation analysis of factors related to ApoB/ApoA-I ratio in patients with anti-NMDAR encephalitis.

	**ApoB/ApoA-I**	
	***R***	***p***
ICU treatment	0.303	0.013^*^
Length of hospital stay	0.244	0.047^*^
CRP	0.398	0.004^*^
ESR	0.407	0.006^*^
Baseline mRS score	0.344	0.004^*^

Only statistically significant factors are presented here. A two-sided *p*-value of <0.05 is defined as statistically significant. anti-NMDAR, anti-N-methyl-D-aspartate receptor; ApoA-I, apolipoprotein A-I; ApoB, apolipoprotein B100; ICU, intensive care unit; CRP, C-reactive protein; ESR, erythrocyte sedimentation rate; mRS, modified Rankin scale.

* *p* < 0.05,

** *p* < 0.001.

### The ratios of the high and low ApoB/ApoA-I groups both improved in the follow-up

**Figure 2:**
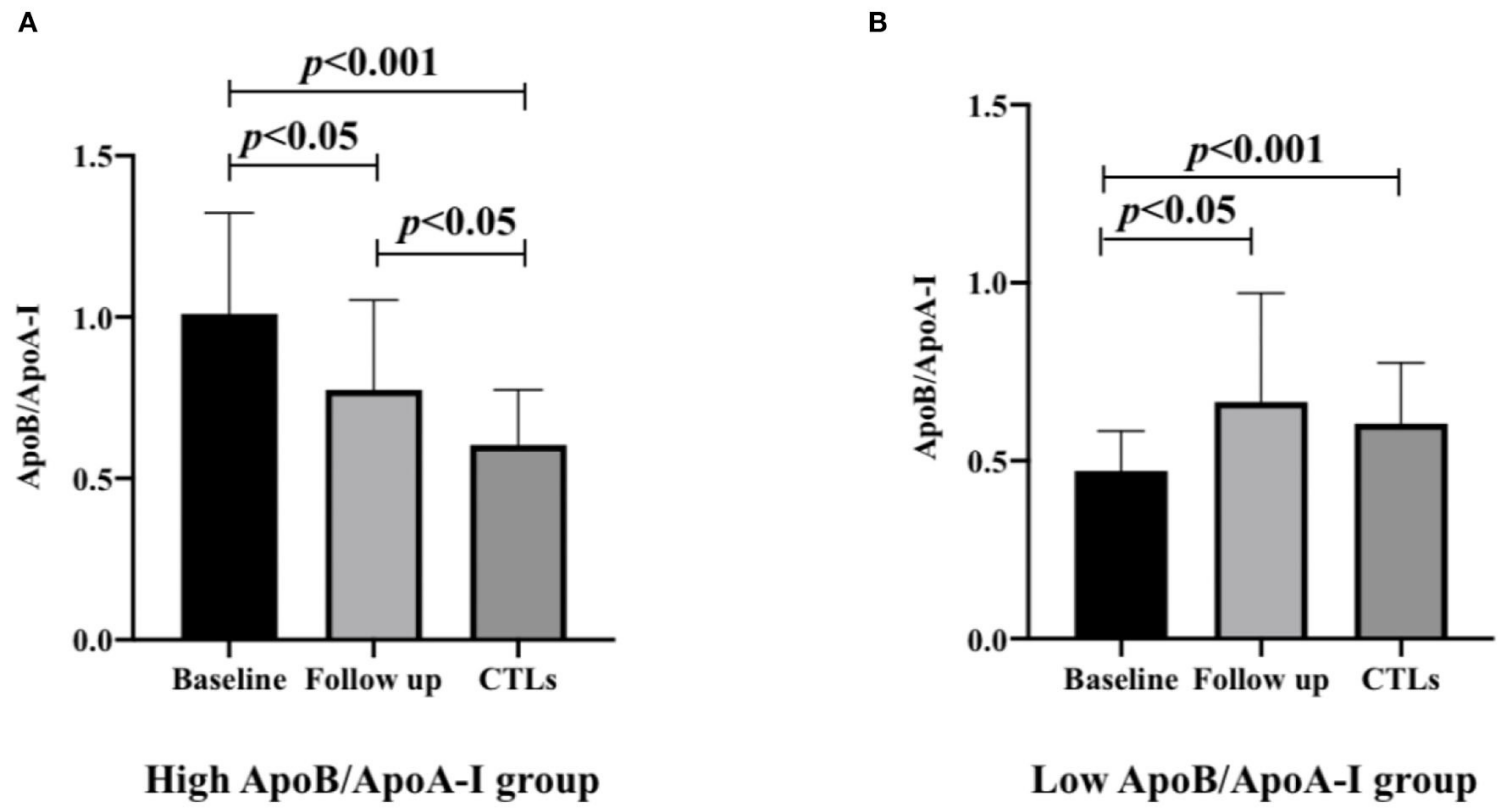
The ApoB/ApoA-I ratios of high and low ApoB/ApoA-I ratio groups in patients with anti-NMDAR encephalitis are compared with CTLs (*n* = 71) at baseline and follow-up. **(A)** High ApoB/ApoA-I ratio group in patients with anti-NMDAR encephalitis. **(B)** Low ApoB/ApoA-I ratio group in patients with anti-NMDAR encephalitis. A one-way analysis of variance (ANOVA) was used for multiple comparisons, followed by the least significant difference (LSD) for *post-hoc* test. A two-sided *p*-value of <0.05 was defined as statistically significant. ApoA-I, apolipoprotein A-I; ApoB, apolipoprotein B100; anti-NMDAR, anti-N-methyl-D-aspartate receptor; CTLs, controls.

In the high ApoB/ApoA-I group, the ratio was significantly elevated in baseline compared to the CTLs (1.00 ± 0.31 vs. 0.60 ± 0.17 *p* < 0.001), and decreased after 3-month follow-up compared to the baseline (0.77 ± 0.28 vs. 1.00 ± 0.31 *p* = 0.003), but still higher than the CTLs (0.77 ± 0.28 vs. 0.60 ± 0.17 *p* = 0.023). In the low ApoB/ApoA-I group, the ratio was significantly decreased at baseline compared to the CTLs (0.46 ± 0.11 vs. 0.60 ± 0.17 *p* < 0.001), but elevated after 3-month follow-up compared to the baseline (0.66 ± 0.31 vs. 0.46 ± 0.11 *p* = 0.027), and had no difference between the follow-up patients and the CTLs (0.66 ± 0.31 vs. 0.60 ± 0.17 *p* = 0.845; Figure 2).

## Discussion

This retrospective study suggests that the increased ApoB/ApoA-I ratio is associated with acute anti-NMDAR encephalitis, but not disease outcomes. Serum ApoB/ApoA-I ratio was related to inflammation and immunity in peripheral blood.

First, the present study found that there were differences in ApoB/ApoA-I, PLT/HDL-C, and neutrophil/HDL-C ratios between the patients with anti-NMDAR encephalitis and CTLs at the baseline. With 3-month follow-up, these three ratios in the patient group changed after treatment. However, logistic regression analysis showed that only ApoB/ApoA-I ratio was closely correlated with severe disease (baseline mRS score of >3). ApoB exists merely in chylomicrons, LDL, VLDL, and IDL, which carryover cholesterol to peripheral tissues (10). ApoA-I is the main apolipoprotein of HDL particles, which can accelerate the outflow of cholesterol from peripheral tissues to the liver and excretion from the liver to bile and feces (10). Variations of the ApoB, ApoA-I, and ApoB/ApoA-I ratio have been confirmed to exert independent effects on the incidence of higher cardiovascular mortality, type 2 diabetes, stroke, and long-term risk of a major cardiovascular event (28–31). Studies have also reported the relationship between these biomarkers and immune-inflammatory diseases. An increase in disease activity of PSA was associated with an increase in ApoB/ApoA-I ratio (21). Patients with systemic lupus erythematosus (SLE) showed a decreased level of ApoA-I (32). In atopic asthma, serum ApoA-I is positively associated with forced expiratory volume in 1 s (FEV1), which was used to assess airflow obstruction, whereas ApoB is correlated with more serious airflow obstruction (33). Anti-NMDAR encephalitis is an autoimmune disease, and its etiology is far from being elucidated (33). Our study found that the serum ApoA concentration decreased, and the level of serum ApoB had no change, but the ApoB/ApoA-I ratio promoted the potential as an indicator of acute disease in patients with anti-NMDAR encephalitis. ApoB accelerates, whereas ApoA-I inhibits the systemic inflammatory state. Thus, an elevated ApoB/ApoA-I ratio probably implied that the pro-inflammatory effect is superior to the anti-inflammatory effect of lipoproteins, so as to indicate the progression of inflammation and disease severity (22). In myocardial infarction, the non-fasting ApoB/ApoA-I ratio has been considered to be a superior biomarker to any other conventional lipid ratio (34). Based on our results, we suggest that the ApoB/ApoA-I ratio might be a key lipid biomarker in anti-NMDAR encephalitis as well.

In patients with anti-NMDAR encephalitis, the subgroup analysis showed that there were many differences between the high and low ApoB/ApoA-I groups in psychiatric symptoms, length of ICU stay, length of hospital stay, serum CRP, serum IgG, serum C3, and ESR. CRP and ESR are inflammatory markers (21), and IgG and C3 mediated immune and inflammatory responses (35). The high ApoB/ApoA-I group patients had significantly more severe psychiatric symptoms, longer length of ICU stay, longer length of hospital stay, and higher levels of inflammatory markers. This suggests that a higher ApoB/ApoA-I ratio may be associated with acute disease and greater activation of inflammatory response. Nevertheless, there was no difference in multiple markers of CSF between the two groups, and the role of the ApoB/ApoA-I ratio might not involve the central nervous system. Another important result of our study is that the ApoB/ApoA-I ratio was positively correlated with ICU treatment, length of hospital stay, baseline mRS score, CRP, and ESR. The elevated ApoB/ApoA-I ratio might more likely indicate the requirement for ICU treatment, longer hospital stay, and higher baseline mRS score in anti-NMDAR encephalitis. The serum ApoB/ApoA-I ratio was related to inflammation and immunity in periphery blood. We observed a change in ApoB/ApoA-I ratio, which largely correlated with the ApoA-I concentration. Several studies have suggested that systemic inflammation is closely related to the levels of serum ApoA-I and ApoB/ApoA-I ratio in both children and adults (36, 37). The levels of serum ApoA-I are decreased in both acute and chronic inflammatory status (38). ApoA-I has been reported to directly inhibit the production of cytokines, so it may play an anti-inflammatory and antioxidant role (38). ApoA-I inhibits monocytes to contact with stimulated T cells, thereby restraining the production of cytokines such as interleukin 1β (IL-1β) and tumor necrosis factor α (TNF-α) (38). ApoA-I downregulates the function of neutrophils, inhibits the inflammatory function of peripheral blood monocytes, and reduces the infiltration of macrophages (38–40). In addition, ApoA-I is related to paraoxonase, an enzyme that can cut down oxidative modification of LDL (41). Moreover, ApoA-I participated in the suppression of complement activation (41). The ApoB/ApoA-I ratio has been reported as well to be related to inflammation and oxidative stress (42). Moreover, it remains unknown whether inflammation is the cause or consequence of serum apolipoprotein levels alterations. There may be a complex and bidirectional relationship between apolipoprotein and inflammation (43). The correlation between ApoB/ApoA-I ratio and inflammatory processes probably interprets the effects of elevated ApoB/ApoA-I ratio on acute anti-NMDAR encephalitis.

In this retrospective study, the ratios of the high and low ApoB/ApoA-I groups both improved in the follow-up. The ratio of the low ApoB/ApoA-I group basically returned to the level of the CTLs, but the high ratio group was still higher than the CTLs. This indicated that the decreased ApoB/ApoA-I ratio is easier to recover after treatment, whereas the elevated ApoB/ApoA-I ratio is difficult to recover. A longer follow-up period or a larger sample size might be required for stratification or subgroup analysis.

The highlight of this study was that the increased ApoB/ApoA-I ratio is associated with acute anti-NMDAR encephalitis, but not disease outcomes. Moreover, the subgroup analysis found that patients with high ApoB/ApoA-I were more serious, which may be related to inflammation and immunity in periphery blood. Therefore, the mechanism of the increased ApoB/ApoA-I ratio of patients with acute anti-NMDAR encephalitis may be related to inflammation and immunity in periphery blood. This may provide a new idea for the study of the mechanism and treatment of anti-NMDAR encephalitis.

The present study has several limitations that should be addressed as well. First, this is a single center, small sample size, and retrospective study, so more centers and larger sample size studies are needed to further explore the relationship between serum ApoB/ApoA-I ratio and anti-NMDAR encephalitis. In addition, the study did not include data related to dead patients with anti-NMDAR encephalitis. The role of the ApoB/ApoA-I ratio in these extremely critical patients needs to be further clarified. Third, the potential impact of treatment is yet a confounding variable. Finally, due to the limitation of the sample size, patients with anti-NMDAR encephalitis are only divided into high and low ApoB/ApoA-I groups for subgroup analysis. More-detailed subgroup analyses may be more conducive to the study of the role of the ApoB/ApoA-I ratio in the disease.

## Conclusion

The present study suggests that the elevated ApoB/ApoA-I ratio is associated with acute anti-NMDAR encephalitis, but not disease outcomes. Serum ApoB/ApoA-I ratio was related to inflammation and immunity in peripheral blood. The findings might provide a new idea for further study in the mechanism and treatment of anti-NMDAR encephalitis.

## Data availability statement

The original contributions presented in the study are included in the article/supplementary material, further inquiries can be directed to the corresponding author/s.

## Ethics statement

The studies involving human participants were reviewed and approved by the Ethics Committee of the Third Affiliated Hospital of Sun Yat-sen University. Written informed consent for participation was not required for this study in accordance with the national legislation and the institutional requirements.

## Author contributions

XC, YL, and XM designed the experiments. YL, LM, and XM collected the data. ZS generated the tables and figures. YL, LM, and DL wrote the manuscript. XC, XM, and ZS revised the manuscript. All authors approved the manuscript.

## Funding

This study was financially supported by the National Natural Science Foundation of China (No. 81971141) and by the Natural Science Foundation of Guangdong Province (No. 2019A1515010201).

## Conflict of interest

The authors declare that the research was conducted in the absence of any commercial or financial relationships that could be construed as a potential conflict of interest.

## Publisher's note

All claims expressed in this article are solely those of the authors and do not necessarily represent those of their affiliated organizations, or those of the publisher, the editors and the reviewers. Any product that may be evaluated in this article, or claim that may be made by its manufacturer, is not guaranteed or endorsed by the publisher.

## Abbreviations

ApoB: apolipoprotein B100
ApoA-I: apolipoprotein A-I
anti-NMDAR: anti-N-methyl-D-aspartate receptor
mRS: modified Rankin scale
ICU: intensive care unit
CRP: C-reactive protein
ESR: erythrocyte sedimentation rate
LDL-C: low-density lipoprotein cholesterol
VLDL: very low-density lipoprotein
IDL: intermediate density lipoprotein
HDL-C: high-density lipoprotein cholesterol
PD: Parkinson's disease
MS: multiple sclerosis
bvFTD: behavioral variant frontotemporal dementia
PSA: psoriatic arthritis
AP: acute pancreatitis
CSF: cerebrospinal fluid
CTLs: healthy controls
MRI: magnetic resonance imaging
CT: computed tomography
CHOL: total cholesterol
TG: triglyceride
IgG: immunoglobulin G
IgA: immunoglobulin A
IgM: immunoglobulin M
C3: complement 3
C4: complement 4
CH50: serum total complement
TBA: tissue-based assay
CBA: cell-based assay
SPSS: Statistical Package for the Social Sciences
SD: standard deviation
ANOVA: analysis of variance
LSD: least significant difference
ORs: odds ratios
CIs: confidence intervals
PLT: platelet
RBC: red blood cell
WBC: white blood cell
SLE: systemic lupus erythematosus
FEV1: forced expiratory volume in 1 s
IL-1β: interleukin 1β
TNF-α: tumor necrosis factor α.
